# Performance evaluation of laboratory professionals on malaria microscopy at health facilities in Bahir Dar city administration, Northwest Ethiopia

**DOI:** 10.1371/journal.pone.0203420

**Published:** 2018-10-18

**Authors:** Kassahun Atalele Jemere, Mulat Yimer Melaku, Tadesse Hailu Jemeber, Megbaru Alemu Abate

**Affiliations:** 1 Felege Hiwot Referral Specialized Hospital, Medical Laboratory Service, Bahir Dar, Ethiopia; 2 Department of Medical Laboratory Sciences, School of Health Sciences, College of Medicine and Health Sciences, Bahir Dar University, Bahir Dar, Ethiopia; Instituto Rene Rachou, BRAZIL

## Abstract

**Background:**

Microscopic diagnosis of Giemsa stained thick and thin blood films by skilled microscopists has remained the gold standard laboratory method for the diagnosis of malaria. However, there is a scarcity of qualified laboratory professionals for correctly diagnosing malaria using microscopy. The aim of this study was to evaluate the performance of laboratory professionals on malaria microscopy at health facilities in Bahir Dar city administration, Northwest Ethiopia.

**Methods:**

A cross-sectional study was conducted from January to March 2017 in Bahir Dar City. A total of 87 laboratory professionals participated in the selected health facilities, with a response rate of (100%). Standardized pre-validated slide panels and questionnaire were distributed to laboratory professionals by the principal investigator. The panel slides were comprised of 5 positives and 3 negative blood films. The laboratory professionals were requested to report the parasite density using semi-quantitative (+) and per micro-liter methods. Their performances of slide readings were compared with the experts’ readings. Agreement in detecting malaria parasites between laboratory professionals and expert was estimated using the kappa score.

**Results:**

The overall sensitivity and specificity of the laboratory professionals in detecting malaria parasites were 83% and 97%, respectively. Similarly, positive predictive values of 98.1% and negative predictive values of 77.7% were reported. The percent agreement between laboratory professionals and expert microscopist in the detection of malaria parasites was 88.5% with a Kappa index of 0.78. Percent agreement in species identification and reporting of Pf/Pv mixed infections were 27.2% and 22.4%, respectively. About 62.2% of the laboratory professionals reported parasite density using semi-quantitative method. While none of them reported per micro-liter method.

**Conclusions:**

The current study showed that laboratory professionals had good performance in parasite detection. However, poor performance was seen in both species identification and reporting of Pf/Pv mixed infections.

## Background

Malaria is a big problem in the world [1]. According to the World Health Organization (WHO) report, malaria infects 212 million and kills 429,000 people throughout the world each year [2]. In Ethiopia, malaria a leading public health problem where an estimated 68% of the population lives in malarious areas [3] Epidemiological and ecological data in Amhara region indicated that more than 75% of the area is malarious [4]. In Ethiopia, two goals have been set to control malaria from 2016–2020; 100% of suspected malaria cases are diagnosed using microscopy and/or Rapid diagnostic test (RDT) within 24 hours of fever onset at health post, health center, and hospital levels and 100% of positive malaria cases are treated according to national guidelines [3]. To achieve these goals, external quality assessment (EQA) on malaria microscopy is a major component. Competency assessment is also a good opportunity to provide continuing education and performance feedback for laboratory professionals and to document valuable objective information for performance evaluations [5].

However, the diagnosis of malaria has still relied on the clinical presentation [6]. Hence, reliable laboratory diagnosis is essential for effective malaria case management, and to evaluate the impact of malaria control interventions [7]. Moreover, laboratory professionals are not always consistent with malaria diagnosis results. For instance, their performance might be affected by the length of time needed for examining a single slide [8], and lack of training and quality control programmers [9]. In addition, quality control (QC) program for malaria microscopy is not adequately developed for evaluation of each laboratory professionals in malaria diagnosis [10].

Thus, data are lacking on laboratory professionals’ performance in detection and identification of malaria parasites in the study area. Therefore, the current study aimed to evaluate the performance of laboratory professionals on detection and identification of *Plasmodium* species using microscopy.

## Methods and materials

### Study design and area

A cross-sectional study was conducted from January to March 2017 on performance evaluation of laboratory professionals on malaria microscopy at health facilities in Bahir Dar city administration. A total of 92 laboratory professionals were working in government hospitals, health centers, and private hospitals. We included 87 laboratory professionals in the study. The remaining five laboratory professionals were excluded from the study since they were not regularly involved in malaria microscopy.

Bahir Dar city is situated on the Southern shore of Lake Tana, having an elevation of 1840 meters above sea level. It has a total population of 221,991 (108,456 men and 113,535 women) [11]. It is one of malaria risk areas in the Amhara Region. There are two government and two private hospitals and ten government health centers. The laboratory service in these facilities provided malaria microscopy on a daily basis.

### Preparation of slide panels

Four ml of venous blood was collected from each of the patients within the age ranges of 20-25years confirmed with for *P*. *falciparum (pf)*, *P*. *vivax (PV)* and *P*. *falciparum/P*. *vivax* (pf/PV) mixed infections. Malaria negative panel slides were prepared from non-malaria infected individuals. Each of the collected blood samples was transferred into EDTA containing glass tubes. The samples were processed within one hour of the collection to preserve leukocyte and parasite morphology. Two experienced validators prepared and validated the panel slides at Amhara Public Health Institution (APHI). Five slides from malaria positive patients and three slides from negative individuals were prepared for thick and thin blood films to be dispatched for each laboratory professionals.

A total of 736 (460 positive and 276 negatives) validated slides were prepared with Pf, Pv and Pf/Pv accounting for 184, 184 and 92 slides, respectively.

For thick and thin blood films preparation, 6 μl and2 μl blood were smeared on a slide. The thick and thin films dried overnight, and the thin films were fixed by dipping in absolute methanol for 10–20 seconds. The slides were stained with 3% Giemsa for 30–45 minutes [2]. Finally, their quality was validated by experienced and certified panel validators and then packed and stored before dispatching.

### Panel slide characteristics

The blood films were, validated and interpreted using three diagnostic criteria: The presence or absence of malaria parasites; Identification of the species and quantification of parasitic load. Then, laboratory professionals were asked to report the detection (*Plasmodium* positive or negative), and identification and quantification of *Plasmodium s*pecies. Slides were considered negative if no *Plasmodium* parasites were found in 100 fields [12]. For parasite density, participants were asked to report the value as asexual parasites/μl [6] and the time spent in examining one slide was allocated [13]. Finally, all slides were arranged in eight sets and then packed with their formats for dispatching to health facilities at Bahir Dar City administration.

### Questionnaire

The principal investigator (PI) pre-tested and standardized the study questionnaire with laboratory professionals at Merawi District Hospital. The questionnaire was designed to collect information about laboratory professionals’ performance. Information collected include: age, sex, educational level, type of health facility, status of in-service training, work experience, the routine practice of the professionals, and a number of slides done per person/per day. The data were collected by the PI.

### Data quality control and management

Strict adherence to the standard operational procedures (SOPs) was assured during slide Preparation. Two validators confirmed the slides. The same microscope mark (CX21) was used to minimize the possible instrumental error. Two validators were participated to confirm the slides. Moreover, the same microscope mark (CX21) was used to minimize the possible instrumental error.

The quality of panel slides was properly checked according to WHO standard before dispatching to the laboratory professionals. Finally, the discordant results were rechecked by the principal investigator, along with other certified laboratory professionals who did not participate in the initial preparation and validation processes.

### Data analysis

Data were entered and analyzed using Statistical Package for Social Sciences (SPSS version 23). Level of performances on detection, species identification and quantification of *Plasmodium* parasites were computed using sensitivity, specificity, percent agreement, kappa, positive and negative predictive value.

### Ethical consideration

Ethical clearance was obtained from Bahir Dar University, College of Medicine and Health Sciences ethical review committee on Ref no: Regional Health Bureau/Research and Technology transfer 1/524/2017 dated on 02/02/ 2017. Then after, permission letters were obtained from Bahir Dar city administration health department. Written informed consent forms were signed by the participating health professionals and blood sample providers. No personal and health facility identifier was included. Individuals were given a unique code number.

## Results

All 87 laboratory professionals included in the study participated. Females accounted for 48 (55.2%) and 60 (69%) of the participants were between 20–30 years of age. Regarding their educational status, 49 (56.3%) were diploma holders. Forty-five (51.7%) of them were working in government health facilities. Of the total 87 laboratory professionals, 77 (88.5%) of them had experiences on malaria diagnoses greater than or equals to two years. More than half, 49 (56.3%) of the laboratory professionals took training on malaria diagnosis (Table 1).

**Table 1:** Socio-demographic characteristics of laboratory professionals at health facilities in Bahir Dar city administration from January to March 2017.

**Characteristic**	**Results**
	N (%)
Age in years	
20–30	60 (69)
31–40	24 (27.6)
>40	3 (3.4)
Total	87 (100)
Sex	
Male	39 (44.8)
Female	48 (55.2)
Total	87 (100)
Educational status	
Diploma	49 (56.3)
Degree	38 (43.7)
Total	87 (100)
Health facilities	
Government	67 (77)
Private	20 (23)
Total	87 (100)
Experience	
<2years	10 (11.5)
≥2years	77 (88.5)
Total	87 (100)
Training	
Yes	49 (56.3)
No	38 (43.7)
Total	87 (100)

**Fig 1:**
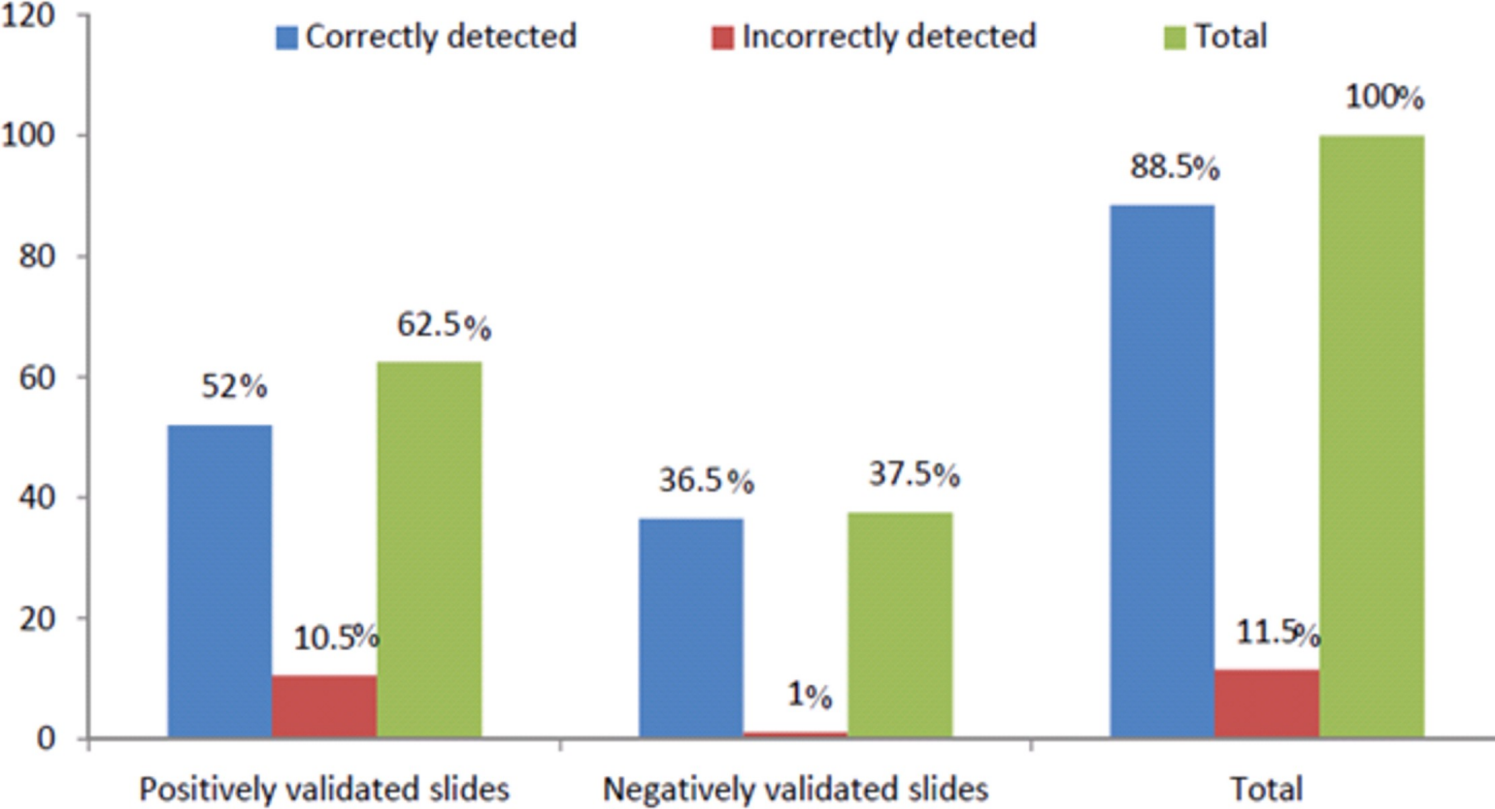
Performance of laboratory professionals participated on detection for *Plasmodium* parasites at health institutions in Bahir Dar city administration from January to March, 2017.

Of the total 696 dispatched slides, 616 (88.5%) were correctly reported. Of the 435 positive slides, 362 (83.2%) were correctly detected as positive and the rest false positive. Of the 261 malaria negative slides, 254 (97.3%) were correctly detected as negative (Fig 1).

**Fig 2:**
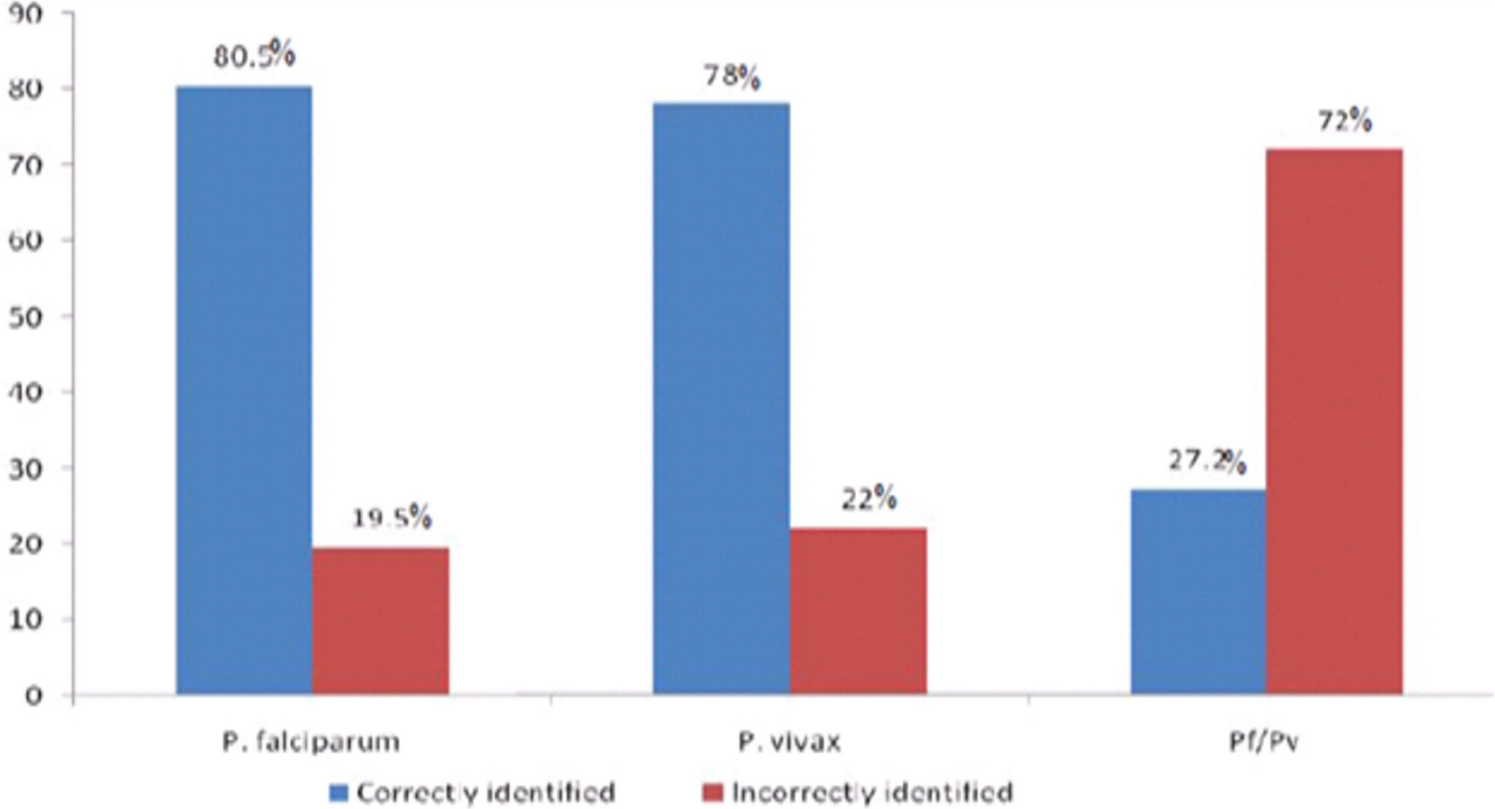
Performance of laboratory professionals on identification of *Plasmodium* species at health institutions in Bahir Dar city administration from January to March, 2017.

Among 362 positively reported slides, the proportion of correct report for Pf, Pv and Pf/Pv mixed infections were 149 (41.2%), 132 (36.5%) and 81 (22.4%), respectively. Moreover, the proportion of correct species identification was 245 (67.7%). Furthermore, the rate of species identification was 120 (80.5%), 103 (78%) and 22 (27.2%) respectively, for Pf, Pv and Pf/Pv mixed infections (Fig 2).

The proportion of correct report of parasite density was 13.4% (23/172) and 18.6% (32/172) for (Pf 1+) and (Pf 4+) Slides. Similarly, the proportion of correct parasite density report in *P*. *vivax* positive slides was 52 (30.2%) slides 18.6% (32/172) in (1+) and 11.6% (20/172) in (3+) slides. However, none of them reported in the micro-liter method (Table 2).

**Table 2:** Performance of laboratory professionals on quantification of parasite density at health facilities in Bahir Dar city administration from January to March 2017.

Laboratory professionals’ results				
Validators’ results		Correct	Incorrect	Total
Parasite density				
Semi-quantitative		N (%)	N (%)	N (%)
Pf (1+)		23 (13.4)	8 (4.7)	31 (18.1)
Pf	(4+)	32 (18.6)	33 (19.2)	65 (37.8)
Pv	(1+)	32 (18.6)	10 (5.8)	42 (24.4)
Pv	(3+)	20 (11.6)	14 (8.1)	34 (19.7)
Total		107 (62.2)	65 (37.8)	172 (100)
Pf/Pv (mixed)		NR	22 (30.1) (	22 (30.1)
Pf		NR	24 (32.9)	24 (32.9)
PV		NR	27 (37)	27 (37)
Total		NR*	73 (100)	73(100)
Total		107(43.7)	138 (56.3)	245(100)

*NR = Not reported

The sensitivity and specificity of laboratory professionals’ on detection of *Plasmodium* parasites were 83% and 97%, respectively, and positive and negative predictive values were 98.1% and 77.7%, respectively. Similarly, sensitivity and specificity of professionals in species identification of *Plasmodium* parasites were 56.3% and 97.3%, respectively. The agreement between the laboratory professionals and validators' on detection and identification of *Plasmodium* parasites were 88.5% (Kappa = 0.78) and 72% (kappa = 0.47), respectively (Table 3).

**Table 3:** Overall sensitivity, specificity, and agreement between the laboratory professionals and validators' on detection and identification of *Plasmodium* parasites at health facilities in Bahir Dar city administration from January to March, 2017.

Laboratory Professionals Results		Validators’ results			Sensitivity	Specificity	PPV	NPV	agreement	Kappa
		Positive	Negative	Total	%	%	%	%	%	
Parasite detection	Positive	362	7	369	83	97	98.1	77.7	88.5	0.78
	Negative	73	254	327						
	Total	435	261	696						
Species identification	Correct	245	7	252	56.3	97.3	97.2	50.9	72	0.47
	Incorrect	190	254	444						
	Total	435	261	696						

PPV = positive predict value. NPV = negative predict value

Laboratory professionals who were working in government health centers had highest performance on the identification of *Plasmodium* species. The overall agreements between the validators’ and laboratory professionals’ performance working at government health centers, government hospitals and private hospitals in identification of *Plasmodium* species were: 67.2% (kappa = 0.4), 83.5% (kappa = 0.69) and 68% (kappa = 0.41), respectively (Table 4).

**Table 4:** Performance of laboratory professionals on the identification of *Plasmodium* species at private and government health facilities in Bahir Dar city administration from January to March 2017.

Species identification by	Laboratory	Validators’ results			Sensitivity	Specificity	Agreement	Kappa
health facilities	professionals result	Positive	Negative	Total		%		
Government Hospitals	Correct	110	3	113	48.9	97.8	67.2	0.4
	Incorrect	115	132	247				
	Total	225	135	360				
Government health centers	Correct	81	0	81	73.6	100	83.5	0.69
	Incorrect	29	66	95				
	Total	110	66	176				
Private Hospitals	Correct	53	4	57	53	93	68	0.41
	Incorrect	47	56	103				
	Total	110	60	160				

From a total of 87 laboratory professionals performances in detection and species identification 37(42.5%) and 6 (6.9%) were graded as expert, 32(36.8%) and 24 (27.7%) reference and 18(20.7%) and 57(65.5%) were labeled as in training, respectively based on WHO classification (Table 5).

**Table 5:** Overall performance of laboratory professionals on *Plasmodium* detection a and species identification with their classification based on WHO recommendation at health facilities in Bahir Dar city administration from January to March 2017.

Parasite detection		Species identification		
Grade	N (%)	Grade	N (%)	
Expert (≥ 90%)	37(42.5)	Expert (≥ 90%)	6(6.9)	
Reference (≥ 80%)	32(36.8)	Reference (≥ 80%)	24(27.6)	
Advanced (≥70%)	0	Advanced (≥70%)	0	
In-Training (<70%)	18(20.7)	In-Training (<70%)	57(65.5)	
Total	87 (100)	Total	87 (100)	

## Discussion

There is evidence for the scarcity of qualified laboratory professionals to correctly diagnose, and report malaria in Ethiopia [4]. This has an impact on malaria case detection, management, prevention, and control programs.

The proportion of correct report 88.5% in this study was consistent with a study done in Hawassa 88% [14] and Zambia 90.3% [15]. However, it was higher than the study conducted in Addis Ababa 71.4% [16]. The relatively higher performance of the laboratory professionals’ compared to Addis Ababa might be attributed to the endemicity of malaria in the current study area which exposes the professionals to malaria microscopy.

The current study showed that 2.7% of the participants reported false positive results. However, it was lower than the study done in Hawassa 6.8% [14], in Tigray 10% [17] and in Democratic Congo 16.7% [18]. This might be due to 88.5% of them had more than two years experienced and 56.5% took more in-service training compared to the study done in Hawassa which was 77% of them had two years experienced and 32.1% took in-service training.

Even if, 56.3% of them took In-service training, yet none of them reported the parasite density as per the WHO guideline. This might be due to poor training quality on malaria microscopy. Thus, the training given on QC for microscopy should target on: Detection of single or mixed *Plasmodium* infections, identification of single or mixed *Plasmodium* species infections, and reporting of the laboratory result as per the WHO guideline.

In the present study, the agreement between the validators and laboratory professionals on detection of *Plasmodium* parasites was 88.5%. This was similar to a study done in Hawassa 88% [14]. But it was higher than the study done in Addis Ababa 71.4% [16] and Hawassa 54% [19].This difference might be due to the fact that a higher proportion of laboratory professionals took training and had more experience in the present study area as compared those laboratory professionals in Hawassa and Addis Ababa.

The overall, sensitivity and specificity of laboratory professionals on detection of *Plasmodium* parasites were 83% and 97%, respectively. Sensitivity and specificity of laboratory professionals were nearly similar to the study conducted in Zambia 88% and 91% [15]. The sensitivity was higher than findings from Tigray 63% [17]. The lower sensitivity in parasite detection might be due to the high proportion of false negative results and hence underreport of true infection in the previous study.

In the current study, overall agreement on the identification of *Plasmodium* species was 72% (kappa = 0.47) and it was defined as ‘moderate agreement’ based on the Kappa index [20]. Laboratory professionals’ performance to identify *P*. *falciparum* and *P*. *vivax* species were nearly similarly 80.5% and 78%, respectively. The overall rate of species identification was consistent with a study conducted in North Gondar 69% [21]. However, it was higher than findings from rechecking laboratories in Ethiopia 64.8% [22] and Addis Ababa 51% [16]. Laboratory professionals showed the lowest performance on species identification in mixed (Pf/Pv) infections 27.2%. Furthermore, identification of Pf/Pv in this study was higher than the study done in Addis Ababa 16.7% [16], Hawassa 20% [19] and in the Peruvian Amazon 17.7% [23]. Of the positively identified slides, 70.2% were reported using semi-quantitative method which is not currently recommended. On the other hand, the remaining 29.8% didn’t report *Plasmodium* species densities at all. These reports were consistent with a study done in Hawassa 28% [19].

The overall agreement of on laboratory professionals’ performance in species identification among government hospitals, health centers, and private hospitals were 67.2% (kappa = 0.40), 83.5% (kappa = 0.69) and 68% (kappa = 0.41), respectively. These results at health centers in this study area were higher than those health centers in Hawassa 69% [14]. It could be an indication that health centers had good delivering of training and EQA.

It is also noted that low sensitivity and specificity of malaria diagnosis were observed in the government and private hospitals as compared to government health centers. This might be due to the Ethiopian health facility referral system. According to this referral system, patients regardless of their cause first visit health centers, then, those patients who were not treated at health centers are referred to hospitals. It is because of this reason that almost all malaria patients who did not develop severe malaria are treated at the health center level. Thus, laboratory professionals who were working at Ethiopian health centers experience in the diagnosis of malaria as compared to hospitals. As a result, more training on malaria microscopy is given at health centers than hospitals.

In this study, 65.5% of laboratory professionals were classified as 'In-training' and 6.9% were at expert level in the identification of *Plasmodium* species. Consistent findings were observed from Addis Ababa as 8.3% were at expert level [16].

### Limitations of the study

In this study performance of laboratory professionals’ in smearing preparation, working solution, staining of blood films, washing habit, and drying conditions for malaria diagnosis were not done.

### Conclusions

The current study showed that laboratory professionals had good performance in parasite detection according to WHO criteria. However, they had poor performance in species and in mixed infections identification. Therefore, EQA should be starting them to fill their gaps.
